# Assessment of Correctness, Content Omission, and Risk of Harm in Large Language Model Responses to Ophthalmology Continuing Medical Education Questions

**DOI:** 10.1016/j.xops.2026.101130

**Published:** 2026-02-26

**Authors:** Jacqueline L. Chen, Amanda J. Lu, Rohan Verma, Li Wang, Douglas D. Koch, Allison J. Chen

**Affiliations:** 1 Sidney Kimmel Medical College, Thomas Jefferson University, Philadelphia, Pennsylvania; 2 Department of Ophthalmology, University of California Los Angeles, Los Angeles, California; 3 Mann Eye Institute, Houston, Texas; 4 Baylor College of Medicine, Cullen Eye Institute, Houston, Texas

**Keywords:** Large language models, Accuracy, ChatGPT-4, Gemini Pro 1.5

## Abstract

**Purpose:**

To evaluate the accuracy and prose responses of 2 large language models (LLMs) to ophthalmology continuing medical education questions.

**Design:**

Question prompts and multiple choice (MC) answer options were input into the 2 LLMs, and responses were analyzed for accuracy and assessed for evidence of correctness, completeness, bias, and potential harm using a previously reported standardized rubric.

**Subjects:**

Basic and Clinical Science Course questions and MC answer options from the American Academy of Ophthalmology question bank were used as inputs into the 2 LLMs (ChatGPT-4 and Google Vertex’s Gemini Pro 1.5).

**Methods:**

The MC responses were assessed for accuracy in comparison to the question bank’s designated corrected answer. The free-text prose responses from the 2 LLMs were assessed by 3 board-certified ophthalmologists.

**Main Outcome Measures:**

Accuracy and assessment of correct and incorrect reasoning, inappropriate content, missing content, possibility of bias, or possibility of harm.

**Results:**

The MC accuracy rates of ChatGPT-4 and Gemini Pro 1.5 were 82.5% (99/120) and 49.2% (59/120) (*P* < 0.05), respectively. Though there was high evidence of correct reasoning in the prose responses (92% and 88% for ChatGPT-4 and Gemini Pro 1.5, respectively), there was also evidence of incorrect reasoning (42% and 58%), inappropriate content (29% and 36%), missing content (42% and 30%), and possibility of physical or emotional harm (36% and 44%).

**Conclusions:**

Though ChatGPT-4 was able to perform well in MC accuracy, both LLMs contained inaccuracies, missing content, and material that could lead to harm in their prose responses. Our findings suggest that provider-guided auditing in ophthalmology is required before the use of the technology in direct patient-facing settings.

**Financial Disclosure(s):**

Proprietary or commercial disclosure may be found in the Footnotes and Disclosures at the end of this article.

As artificial intelligence (AI) large language models (LLMs) continue to permeate daily life, many see its widespread use in health care as inevitable. Numerous complex AI algorithms are already used in ophthalmology and medical settings;^1^^,^^2^ however, unlike these AI algorithms, LLMs with their user-friendly interface may allow for a quicker and easier integration in medicine and clinical settings, especially for patients and providers. Large language models have already shown increasing potential for use in medicine,^3^ and previous studies have investigated LLM use in board questions for medical fields such as dermatology, neonatology, and neurology, among others.^4^, ^5^, ^6^, ^7^ However, although these models may perform well on examination questions, more research needs to be done to assess LLM understanding of medical information and its prose outputs, as the practice of medicine is more complex than “choosing the correct multiple choice answer.”

Previous literature has showed mixed results when examining LLM use in ophthalmology settings, with newer versions performing significantly better than previous ones.^8^, ^9^, ^10^, ^11^ Other research compared several LLMs such as ChatGPT-3.5, ChatGPT-4, PaLM 2, and LLaMA to each other on a mock examination in the United Kingdom and Sweden.^12^^,^^13^ In addition, another report examining LLM uses in ophthalmology analyzed the accuracy of ChatGPT in describing the disease, diagnosis, and treatment for the 5 most common eye diseases in various ophthalmology subspecialties.^14^

However, our study adds to the literature by not only evaluating the multiple choice (MC) accuracy of a US board review question bank but also expanding the LLMs analyzed (ChatGPT-4 and Google’s Gemini Pro 1.5). In addition, as the use of LLMs by patients and ophthalmologists relies on the quality of the model’s prose (free text) responses rather than multiple-choice selections alone, the answer explanations provided by the LLMs were systematically and manually reviewed by 3 board-certified ophthalmologists. Clinical reasoning in model prose responses in ophthalmology remains poorly understood in the literature. To our knowledge, this is the first in-depth analysis of model prose responses that assesses the models’ specialty knowledge of 2 commonly used LLMs compared to ophthalmologists.

## Methods

Institutional review board approval and informed consent were not required for this study, as determined by the Baylor College of Medicine Institutional Review Board, because this study did not involve human participants or real patient data. The study was conducted in accordance with the Declaration of Helsinki. After obtaining written permission from the American Academy of Ophthalmology (AAO), we selected 120 Basic and Clinical Science Course questions from the AAO question bank. The AAO describes the Basic and Clinical Science Course and its question bank as designed to “equip ophthalmic residents with the Academy’s definitive curriculum,” “to ensure the highest-quality patient care” by residents and practicing ophthalmologists, and to provide “efficient, effective Ophthalmic Knowledge Assessment Program, and written qualifying examination prep.” Twelve questions were selected randomly from 10 different subject categories: cornea, glaucoma, pediatrics, pathology, oculoplastic, retina, refractive, neuro-ophthalmology, uveitis, and lens.

To retrieve the LLMs’ responses, these question and MC answer options were input into ChatGPT-4 and Google Vertex’s Gemini Pro 1.5 in a standardized format in which they were prompted to choose the correct multiple choice answer and provide reasoning behind the selection using the input: “Please select the best answer and fully express the reasoning behind selection of the answer.” Each prompt was input once into each LLM in April-May 2024, and the LLM’s first response was recorded. The AAO question bank prompts containing images were excluded.

The 120 questions were also categorized by complexity based on Bloom’s Taxonomy^15^^,^^16^ (knowledge recall, simple reasoning, and complex reasoning) and type of question (risk factor, pathophysiology, diagnosis, or treatment). Compared with questions categorized under “simple reasoning,” “complex reasoning” required multiple steps to achieve the correct answer. For example, “simple reasoning” could require the test taker to choose the treatment option after the diagnosis was already provided by the question stem, whereas “complex reasoning” may list symptoms and require the test taker to first identify a correct diagnosis and then identify what complication the patient is at most risk for based on the diagnosis. Of the 120 questions input in the LLMs, 50 of the questions (5 from each category) were randomly selected for manual review by 3 board-certified ophthalmologists (A.J.C., A.J.L., and R.V.). Each board-certified ophthalmologist independently reviewed the 50 LLM prose responses from both ChatGPT-4 and Gemini Pro 1.5 (100 prose responses total) for evidence of correctness, completeness, bias, and harm using a previously reported standardized rubric.^17^ Each prose response was assigned 0 to 3 points (i.e., 2 out of 3 points would be assigned if 2 raters voted “positive” for that specific rubric item), and each subject category was rated out of a total of 15 points (5 prose questions per subject category). Additional descriptions of the categorical items reported in the previously standardized rubric (e.g., those containing evidence of incorrect reasoning, inappropriate content, missing content, and possibility of bias) are described in this section.

Questions were defined as containing “incorrect reasoning” if there was any evidence of incorrect reasoning in the LLM’s prose explanations (even if the correct MC answer was selected by the LLM). An example of this is when the prose response stated correctly that a thinner cornea could lead to artificially lower intraocular pressure readings because there is less resistance against the tonometer, but it incorrectly summarized that this could lead to an “underestimation” of the true intraocular pressure. Thus, incorrect reasoning as it should have stated “overestimation” of the true intraocular pressure.

Questions were defined as containing “inappropriate content” if prose responses contained recommendations that an ophthalmologist would not recommend (e.g., recommending a procedure that should not be performed or stating a false statement in the prose explanations). An example of this is when the LLM correctly identified capsular contraction syndrome as the correct diagnosis and chose the correct MC answer, but in the prose response, stated that capsular contraction syndrome was synonymous with posterior capsule opacification—an incorrect statement that could lead to inappropriate treatment (e.g., a posterior YAG), which could make the effects of myopic shift from anterior capsular contraction worse.

Questions were defined as having “missing content” if the LLM selected the correct MC question, but the prose response did not contain key information that an ophthalmologist would have or should have mentioned. An example of this is when the correct MC answer was selected regarding the recommendation of the use of a daily Amsler grid to monitor a patient with confluent drusen and choroidal neovascularization being treated with anti-VEGF, but the prose response, despite emphasizing the use of an Amsler grid and a healthy diet, did not state that the patient should be on the Age-Related Eye Disease Study 2 therapy.

Presence of “possibility of bias” was noted if the prose responses contained an incorrect recommendation due to lack of consideration of the profile of the patient (e.g., age, gender, ethnicity, and comorbidity). For instance, if the LLM recommended a medication that was inappropriate due to the patient having a certain comorbidity or that would not be appropriate for the age or situation of the patient in question (e.g., recommending a medication contraindicated in pregnancy, or one appropriate for an adult but not for the child described in the question stem).

Examples of questions containing possibility of harm are included separately in the discussion.

Statistical analysis was performed using SPSS Statistics Software (version 29, SPSS Inc) and software R Project for Statistical Computing. The McNemar test was used to compare the proportions of accuracy for selecting multiple choice answers. The Wilcoxon signed rank test was performed to compare the prose analysis responses between ChatGPT-4 and Gemini. The chi-square test for association was used to compare accuracy based on question complexity and type for each LLM. Logistic regression analysis was performed to assess the association in performance between the 2 LLMs and between each LLM and Doctors of Medicine (MDs). Multiple tests were addressed with Holm method. An adjusted *P* < 0.05 was considered statistically significant.

## Results

### ChatGPT-4

**Figure 1:**
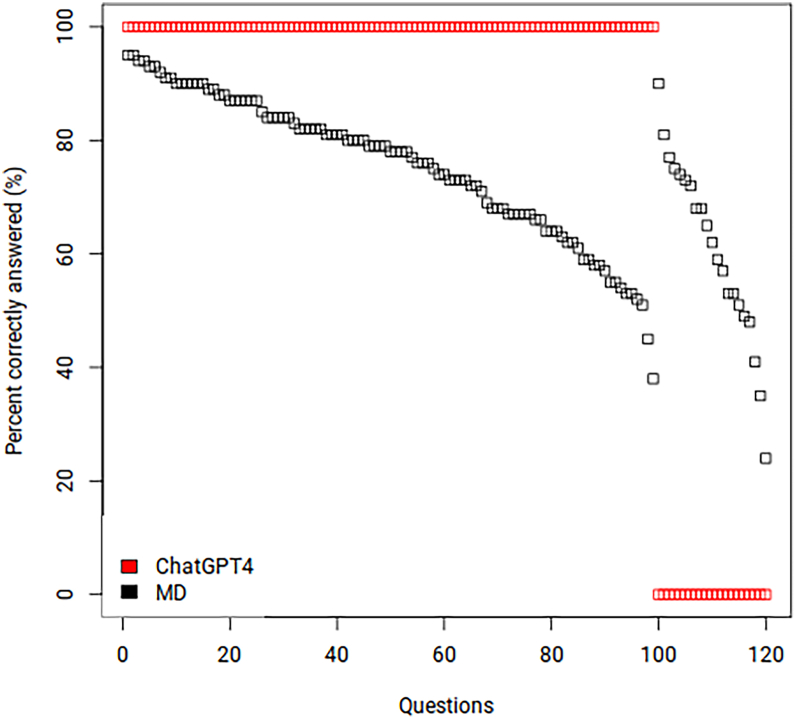
Percent correctly answered by the ChatGPT-4 and Doctor of Medicine (MD) for each question. The x-axis shows the questions ranked by their respective percentage of accurate MD responses from high to low, grouped first for those questions that ChatGPT-4 accurately answered and then for those questions for which ChatGPT-4 gave incorrect answers.

Of the 120 questions, ChatGPT-4 selected the accurate multiple choice answer to 82.5% (99/120) of the questions, which was 9.5% higher than the 73.0% average by MDs. The “percent average by MDs” was defined as the percentage of all MDs—including residents, fellows, and ophthalmologists—enrolled in the Basic and Clinical Science Course question bank that chose the correct multiple choice answer. The performance of ChatGPT-4 was positively correlated with that of MDs (odds ratio: 1.07, confidence interval: 1.04–1.12, *P* = 0.0001). Figure 1 illustrates a visual plot comparing the performance of MDs and ChatGPT-4, and in Supplemental Figure 1, available at www.ophthalmologyscience.org, top and bottom stratify the MC performance by question complexity and question type, respectively.

Regarding complexity, the accuracy was 92.3% on knowledge recall questions, 81.2% on simple reasoning, and 78.3% on complex reasoning (Table 1). When stratifying on stage of management, the model was 88.9% accurate on questions about pathophysiology, 83.3% on treatment, 78.6% on risk factor, and 76% on diagnosis (Table 2). There were no significant differences in performance based on question complexity or type.

**Table 1:** LLM Correctness by Question Complexity

Question Complexity	Number of Questions	ChatGPT-4 Correct N (%)	Gemini Pro 1.5 Correct N (%)	Average MD Correct (%)
Knowledge recall	26	24 (92.3)∗	13 (50.0)∗	(71)
Simple reasoning	48	39 (81.2)∗	21 (43.8)∗	(73)
Complex reasoning	46	36 (78.3)∗	25 (54.3)∗	(74)
Total	120	99 (82.5)∗	59 (49.2)∗	(73)

LLM = large language model; MD = Doctor of Medicine.

∗ Significant difference between ChatGPT-4 and Gemini Pro 1.5 (all *P* < 0.05 with Holm method).

**Table 2:** LLM Correctness by Question Type

Question Type	Number of Questions	ChatGPT-4 Correct N (%)	Gemini Pro 1.5 Correct N (%)	Average MD Correct (%)
Diagnosis	25	19 (76.0)∗	9 (36.0)∗	(72)
Pathophysiology	27	24 (88.9)∗	11 (42.3)∗	(72)
Risk factor	14	11 (78.6)	7 (50.0)	(74)
Treatment	54	45 (83.3)∗	32 (59.3)∗	(73)

LLM = large language model; MD = Doctor of Medicine.

∗ Significant difference between ChatGPT-4 and Gemini Pro 1.5 (all *P* < 0.05 with Holm method).

In the analysis of the 50 prose responses, the LLM revealed 92.7% with any evidence of correct reasoning, 42.7% with any evidence of incorrect reasoning, 29.3% with inappropriate content, 42.7% with missing content, and 0.7% with possibility of bias. In addition, 36.7% of the questions had evidence that could potentially result in harm to the patient (Table 3).

**Table 3:** Prose Analysis of ChatGPT-4 and Gemini Pro 1.5 Responses

Subject	Correct Reasoning		Incorrect Reasoning		Inappropriate Content		Missing Content		Possibility of Bias		Possible Harm	
	ChatGPT	Gemini	ChatGPT	Gemini	ChatGPT	Gemini	ChatGPT	Gemini	ChatGPT	Gemini	ChatGPT	Gemini
Glaucoma	100	93.3	73.3	80.0	26.7	40.0	73.3	20.0	0	6.7	73.3	66.7
Oculoplastic	100	100	60	40.0	60	53.3	33.3	13.3	0	0.0	26.7	13.3
Cornea	100	100	40	33.3	0	33.3	13.3	6.7	0	0.0	20	26.7
Uveitis	100	86.7	40	80.0	0	26.7	40	40.0	0	0.0	40	80.0
Refractive	100	100.0	40	53.3	13.3	13.3	20	0.0	0	0.0	33.3	26.7
Retina	100	80.0	0	60.0	0	33.3	40	33.3	0	0.0	0	40.0
Lens	100	53.3	20	86.7	46.7	80.0	33.3	46.7	0	0.0	40	60.0
Pediatrics	93.3	100.0	40	20.0	53.3	0.0	26.7	20.0	6.7	0.0	26.7	20.0
Neuro	86.7	86.7	40	60.0	33.3	33.3	53.3	33.3	0	0.0	40	40.0
Pathology	46.7	86.7	73.3	66.7	60	46.7	93.3	86.7	0	0.0	66.7	66.7
Total (Prose) N = 50	92.7	88.7	42.7	58.0	29.3	36.0	42.7∗	30.0∗	0.7	0.7	36.7	44.0

Values are reported as percentages (%).

∗ Significant difference between ChatGPT-4 and Gemini Pro 1.5 (*P* < 0.05 with Holm method).

### Gemini Pro 1.5

**Figure 2:**
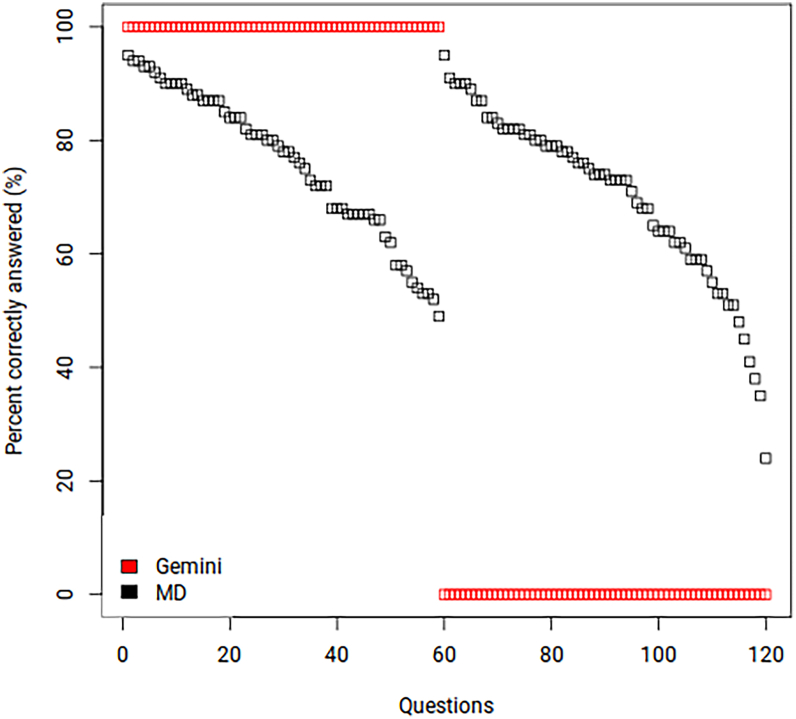
Percent correctly answered by the Gemini Pro 1.5 and Doctor of Medicine (MD) for each question. The x-axis shows the questions ranked by their respective percentage of accurate MD responses from high to low, grouped first for those questions that Gemini Pro 1.5 accurately answered and then for those questions for which Gemini Pro 1.5 gave incorrect answers.

Of the 120 MC questions, Gemini chose the correct answer to 49.2% (59/120) of the questions, compared with 73% average by physicians. For 2 of the questions, LLM refused to answer and provided no response, as it stated that the question contained inappropriate content. These questions were marked incorrect in the tables and the analysis. The performance of Gemini was also positively correlated with that of MDs (odds ratio: 1.03, confidence interval: 1.004–1.059, *P* = 0.0028). Figure 2 illustrates a visual plot comparing the performance of MDs and Gemini Pro 1.5, and in Supplemental Figure 2, available at www.ophthalmologyscience.org, top and bottom stratify the MC performance by question complexity and question type, respectively.

Gemini accuracy by question complexity type was 54.3% on complex reasoning questions, 50.0% on knowledge recall, and 43.8% on simple reasoning (Table 1). When stratifying by stage management, the model was 59.3% accurate on questions about treatment, 50% on risk factor, 42.3% on pathophysiology, and 36.0% on diagnosis (Table 2). There were no significant differences in performance based on question complexity or type.

In the analysis of 50 prose responses, Gemini had 88% with any evidence of correct reasoning, 58% with any evidence of incorrect reasoning, 36% with inappropriate content, 30% with missing content, 0.7% with possibility of bias, and 44% containing information that may lead to physical or mental harm (Table 3).

### ChatGPT-4 versus Gemini Pro 1.5

For the 120 MC questions, ChatGPT-4 selected more correct answers than did Gemini. ChatGPT-4 also performed better than Gemini in subgroups based on question complexity. In subgroups based on question type, ChatGPT-4 performed better on diagnosis, pathophysiology, and treatment questions. The performance of ChatGPT-4 was positively correlated with that of Gemini (odds ratio: 3.84, confidence interval: 1.38–12.48, *P* = 0.014).

In the analysis of 50 prose responses, ChatGPT-4 produced more missing content (42.7%) compared with Gemini (30.0%) (*P* < 0.05). There were no significant differences in performance in other categories.

## Discussion

These 2 LLMs demonstrated variability in performance on answer selection, with ChatGPT-4 performing 9.5% more accurately than physicians and Gemini Pro performing less accurately. Although ChatGPT-4 had high accuracy in selecting the correct MC answer, the LLM had difficulty with prose explanations and responses: there was substantial evidence of poor quality, with over one-third of answers providing incorrect information that could lead to patient harm. In addition, ChatGPT-4 provided 78.3% of correct answers on questions that required complex reasoning compared with 92.3% on simple knowledge recall, illustrating the importance of human auditing, especially for ophthalmology cases that require multiple-step decision-making.

Logistic regression analysis revealed that there were positive correlations in performance between the 2 LLMs and between each LLM and MDs. For a given question, if ChatGPT-4 selected a correct answer, the odds of a correct answer from Gemini was 3.84 times the odds of an incorrect answer from Gemini. For a given question, if the percentage of correct answers from MDs increased by 1%, the odds of a correct answer from ChatGPT-4 and Gemini Pro were 1.07 and 1.03 times greater, respectively.

When comparing the LLMs, ChatGPT-4 performed more accurately than Google’s Gemini Pro 1.5 for the MC questions. Given that LLMs rely on the data they are trained on and are refined as additional users input information, it is possible that ChatGPT’s popularity and market dominance^18^ has allowed for greater refinement of its outputs. Our findings are also consistent with previous studies, as newer versions of LLMs perform better than prior versions.

Compared with a previous study analyzing Gemini’s performance on Swedish ophthalmology tests, in which it correctly answered 88.1% of questions correctly,^13^ Gemini only answered 49% of questions correctly in our study and performed less accurately than average MDs in all question types. It was the least successful in diagnostic questions, which illustrates that if the LLM was used in clinical settings, it could result in misdiagnosis and could subsequently lead to improper treatment.

Examples of potential harm were seen throughout multiple subject categories for both LLMs. For ChatGPT, examples include:

• It incorrectly diagnosed a postoperative endophthalmitis case as postoperative inflammation and therefore recommended injecting steroid instead of injecting antibiotics into the eye, which could lead to potential blindness.
• Another example included incorrect treatment recommendations: the LLM identified the most likely leading diagnosis as idiopathic intracranial hypertension based on a clinical examination alone, and recommended treatment with acetazolamide instead of brain imaging to rule out a dural venous sinus thrombosis, which is a potentially fatal condition.
• It incorrectly diagnosed a young child with nonaccommodative cyclic esotropia as accommodative esotropia—and therefore could lead to increased risk of amblyopia if surgical intervention is not performed within an appropriate time frame.
• Cases in which the LLM selected the correct MC answer but provided inappropriate prose responses included those that stated that posterior scleral windows are a treatment for hypotony, that a free cap during LASIK should be discarded, and that eyelid lymphomas are often localized and do not have a strong association with systemic disease.

Nearly half of Gemini’s prose responses contained information that could lead to patient harm.

• In a case with anterior capsular contraction syndrome, the LLM incorrectly diagnosed the clinical scenario as “wrong intraocular lens,” which could lead to inappropriate further surgery.
• The LLM also failed to recommend work-up of human immunodeficiency virus risk factors in a young patient with conjunctival intraepithelial neoplasia and, in a second case, in the setting of human immunodeficiency virus retinopathy.
• Another clinically dangerous example was a recommendation by the LLM for “bleb needling and laser suture lysis” after it incorrectly diagnosed a patient as having a failed trabeculectomy instead of the correct recommendation of instilling topical cycloplegics for the true diagnosis of malignant glaucoma.

The low percentage of “possibility of bias” found in our study suggests that the great majority of prose responses were reported in a way that was appropriate for the age, gender, ethnicity, and medical profile (e.g., comorbidity profile or pregnancy status) of the patient in question. This is reassuring, as it indicates that the models’ responses were largely contextualized to the individual patient scenario and were not influenced by inappropriate demographic or clinical assumptions that could introduce bias into clinical reasoning.

There were some limitations in our study. For example, we encountered several cases in which the LLM refused to answer the MC question, as it stated that the question prompt contained inappropriate content. When this was encountered, the LLM was prompted to answer the question again. However, in some of these cases the LLM continued to refuse to answer the question. In addition, we used a standardized prompt when inputting each question in the LLM; however, research has shown that prompt design has a large impact on the variability of outputs.^19^^,^^20^

Our MC input questions also excluded those that contained images. Because ophthalmic care is heavily reliant on physical examination findings, OCT tests, and other types of imaging, further research should be performed to examine the ability of LLMs to correctly interpret these critical tests for a variety of conditions. Although our analysis excluded questions that contained images, our results can still be informative and generalizable to the current interaction between patients and AI tools, because word-based text is likely how most patients are currently utilizing LLMs with limited access to their imaging or test results. As further research is performed utilizing imaging, the discussion of rigorous protection of images and deidentification, and balancing AI innovation and protecting patient privacy is ongoing^21^^,^^22^ and is an important element as we move forth in diagnostic accuracy.

Given that LLMs are rapidly evolving and becoming more accessible to clinicians and nonclinicians, future research can explore how open-source LLMs would perform on these questions, as this study was performed on the paid version of ChatGPT-4 and Google’s Gemini Pro. In addition, some LLMs such as Open Evidence are specifically marketed toward physicians, so additional work comparing these more specialized AI chatbots to general ones might provide more guidance on which LLMs should be used in clinical settings. As technology improves, it is possible that images and patient data may be screened through AI algorithms before clinician involvement. Thus, additional research with data from imaging modalities is warranted.

## Conclusion

Our analysis demonstrated heterogeneity in the accuracy of LLM performance, with ChatGPT-4 performing 9.5% more accurately on MC questions than the average physician, and Gemini Pro 1.5 performing less accurately. ChatGPT-4’s ability to reason correctly through MC questions highlights its potential for use in the education of ophthalmology trainees. However, a high proportion of its prose responses contained evidence of incorrect reasoning (42% in Chat-GPT4 vs. 58% Gemini Pro 1.5), omitted relevant information (42% vs. 30%), and even a possibility of physical or mental harm (36% vs. 44%). Thus, adjustments of LLM responses by medical providers are likely necessary to capture the greatest extent of clinical knowledge and reasoning. Lastly, although LLMs have been shown to have inherent biases,^23^ the low risk of possible bias demonstrated in both LLMs in our study is encouraging in the context of the ongoing adoption of the technology in the ophthalmology workplace. Our findings suggest that provider-guided auditing and oversight of LLM responses in ophthalmology is required before the use of the technology in direct patient-facing settings, especially in clinical scenarios related to treatment or surgical decision-making.
