# Supplementary material for: Assessment of Correctness, Content Omission, and Risk of Harm in Large Language Model Responses to Ophthalmology Continuing Medical Education Questions
Source: Ophthalmol Sci. 2026 Feb 26;6(5):101130. doi: 10.1016/j.xops.2026.101130 (PMC13019321; doi:10.1016/j.xops.2026.101130)

Supplemental Figure 1: Percent correctly answered by the ChatGPT-4 and MD for each question in subgroups analyzed by question complexity (top) and question type (bottom). The x-axis shows the questions ranked by their respective percentage of accurate MD responses from high to low, grouped first for those questions that ChatGPT-4 accurately answered and then for those questions for which ChatGPT-4 gave incorrect answers.

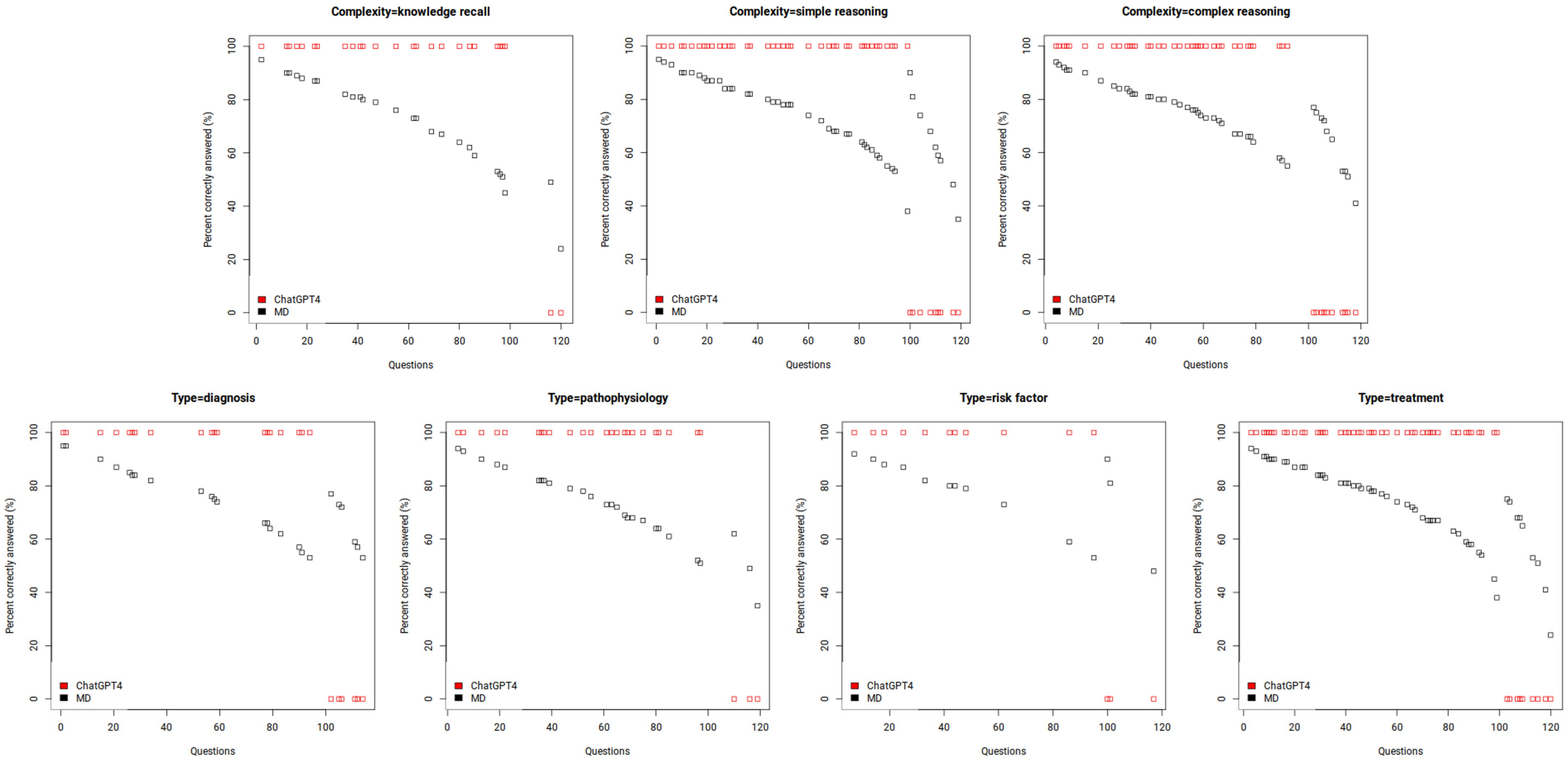

Supplement: Supplemental Figure 1 [file mmc1.pdf]
